# Relationship between IGF-I Concentration and Metabolic Profile in Children with Growth Hormone Deficiency: The Influence of Children's Nutritional State as well as the Ghrelin, Leptin, Adiponectin, and Resistin Serum Concentrations

**DOI:** 10.1155/2017/5713249

**Published:** 2017-05-15

**Authors:** Renata Stawerska, Joanna Smyczyńska, Maciej Hilczer, Andrzej Lewiński

**Affiliations:** ^1^Department of Endocrinology and Metabolic Diseases, Polish Mother's Memorial Hospital-Research Institute, Rzgowska Street 281/289, 93-338 Lodz, Poland; ^2^Department of Pediatric Endocrinology, Medical University of Lodz, Rzgowska Street 281/289, 93-338 Lodz, Poland; ^3^Department of Endocrinology and Metabolic Diseases, Medical University of Lodz, Rzgowska Street 281/289, 93-338 Lodz, Poland

## Abstract

**Background:**

Some, however not all, children with growth hormone deficiency (GHD) reveal a tendency towards metabolic disorders. Insulin-like growth factor I (IGF-I) is the main mediator of GH anabolic effects.

**Objective:**

The aim of the study was to compare ghrelin, adiponectin, leptin, resistin, lipid, glucose, and insulin concentrations in GHD children, depending on the IGF-I bioavailability.

**Methods:**

The analysis comprised 26 children with GHD, aged 5.7–15.3 yrs. Fasting serum concentrations of IGF-I, IGFBP-3, ghrelin, leptin, adiponectin, resistin, lipids, glucose, and insulin were measured. The GHD children were divided into two subgroups: (1) with lower IGF-I/IGFBP-3 molar ratio and (2) with higher IGF-I/IGFBP-3 molar ratio. The control group consisted of 39 healthy children, aged 5.1–16.6 yrs, of normal height and body mass.

**Results:**

GHD children with lower IGF-I/IGFBP-3 molar ratio were found to have a significantly lower body mass and insulin and triglyceride concentrations, as well as significantly higher ghrelin and adiponectin concentrations than GHD children with higher IGF-I/IGFBP-3.

**Conclusions:**

A better metabolic profile characterised GHD children with low IGF-I bioavailability. This phenomenon may be the result of high adiponectin and ghrelin concentrations in those children and their influence on adipose tissue, glucose uptake, and orexigenic axis.

## 1. Introduction

It is well known that growth hormone (GH) promotes linear growth during childhood. However, GH also displays considerable metabolic activity: it stimulates lipolysis with an elevation of circulating free fatty acids, as well as promotes gluconeogenesis and reduces peripheral glucose uptake, which is the reason of relative insulin resistance [1, 2]. Thus, adult patients with isolated GH deficiency (GHD) display unfavourable lipid profile with increased fat mass, as well as increased insulin secretion, though with good insulin sensitivity [1, 3]. However, not all children with GHD show a tendency towards metabolic disorders [4–8].

The main peripheral mediator of GH activity is IGF-I, and GHD is defined as the secondary IGF-I deficiency. It should be noted that—besides the low IGF-I serum concentration—an important role is played by the decreased IGF-I bioavailability. Both the IGF-I bioavailability and the stability of its concentration are determined by binding to specific proteins, especially IGFBP-3. The IGF-I/IGFBP-3 molar ratio is considered to be the IGF-I bioavailability index [9].

Recently, we have observed a negative correlation between IGF-I/IGFBP-3 molar ratio and ghrelin concentration in short stature children [10]. Thus, we have suggested that the lower bioavailability of IGF-I is a stimulating factor for the ghrelin synthesis in a compensatory mechanism. We also proved that ghrelin concentrations were significantly higher in children with GHD when compared to children with idiopathic short stature (ISS) and to children with normal height [11]. Ghrelin—the peptide hormone produced in the stomach—is a natural endogenous ligand for the GH secretagogue receptor [12]. In addition to its role as a stimulator of GH release, it is the most potent known endogenous orexigenic peptide that regulates appetite and body weight [13, 14]. Ghrelin reduces lipolysis, and it directly promotes weight gain, fat accumulation, and adipogenesis, mainly in a GH-independent way [15]. Moreover, it reduces insulin secretion and induces hyperglycaemia [16].

Futhermore, an important target for GH and IGF-I action is adipose tissue. Adipocytokines produced by adipose tissues, such as leptin, adiponectin, and resistin, are also significant factors in regulating fatty acid metabolism, as well as insulin secretion and sensitivity.

Leptin is strongly correlated with the body mass [17], and at the level of the hypothalamus, it inhibits the appetite; thus, its action is opposed to ghrelin action [18]. Leptin also increases hepatic gluconeogenesis and muscle fatty acid oxidation [19]. Moreover, it plays atherogenic, prothrombotic, and angiogenic roles by stimulating vascular inflammation, oxidative stress, and smooth muscle hypertrophy, contributing to pathomechanism of hypertension and atherosclerosis [20]. In children with GHD, the results of leptin concentrations are divergent [7, 8, 21].

It is thought that adiponectin, similar to GHRH, induces GH secretion, and the high adiponectin levels in a model of GHRH resistance are described as a compensatory mechanism to the lack of GHRH action [22]. Adiponectin stimulates uptake and glucose utilization, as well as fatty acid oxidation while it inhibits gluconeogenesis [23]. The effect is a reduction of free fatty acid and triglyceride concentrations and an increase in insulin sensitivity, manifested by a decrease in blood glucose levels without increasing insulin levels [20, 23].

Resistin is known to be a mediator of GH-modulated insulin sensitivity, and in untreated GHD children, higher resistin levels have been observed [24]. It is likely that resistin is linked to obesity and insulin resistance [8].

In the present study, we have decided to analyse the metabolic profile (insulin resistance and lipids) in two untreated groups of GHD patients: (1) with lower (worse) IGF-I bioavailability and (2) with higher (better) IGF-I bioavailability, and we have tried to evaluate the additional role of nutritional state, as well as ghrelin, leptin, adiponectin, and resistin on metabolic profile in these groups.

## 2. Materials and Methods

The study group consisted of 26 short children (11 girls and 15 boys) aged 5.7 to 15.3 yrs (the mean age ± SD: 10.91 ± 2.55 years) in whom isolated GHD was diagnosed, based on the following criteria:

1. Height standard deviation score (HSDS) below −2.0 from the mean value for child's age and sex [25] (children's height was measured using a stadiometer)
2. Low height velocity below −1.0 SD from the mean value for child's age and sex
3. Excluding genetic reasons of the short stature (i.e., Turner's syndrome, Prader-Willi syndrome)
4. Excluding untreated hypothyroidism, chronic diseases, or undiagnosed gastrointestinal tract complaints
5. Decreased GH secretion: GH_max_ values below 10 ng/ml during a 3-hour nocturnal profile and during two stimulation tests: the first one, after clonidine administered orally (with the dose of 0.15 mg/m^2^ of the body surface) and GH concentration measurements at time 0 and at 30th, 60th, 90th, and 120th minutes of the test and the second one, after intramuscular administration of glucagon (in the dose of 30 *μ*g/kg of body weight, not exceeding 1 mg), with GH concentration measurements at time 0 and at 90th, 120th, 150th, and 180th minutes
6. Normal concentration of other pituitary hormone secretion—excluding multiple hormonal pituitary deficiency (MPHD)
7. Normal results of MR images of the hypothalamic-pituitary region.

In all the children, on the faith of the child's current position on percentile charts, the height age (HA) was calculated (as the age ascribed to the 50th percentile for a given child's height). The body mass was assessed in all patients and that was followed by calculation of the body mass index standard deviation score for chronological age (BMI SDS for CA) and for height age (BMI SDS for HA). The stage of puberty was assessed in each child by Tanner's scale. In all the children, we confirmed the Tanner I (prepubertal) stage. It was related to the delayed bone age (which was assessed based on X-rays of nondominant hand and wrist, according to Greulich-Pyle's standards): below 11 years in girls and below 12 years in boys in each of our GHD child, and, in consequence, the delay of biological development).

Moreover, fasting serum concentrations of total ghrelin, leptin, adiponectin, resistin, lipids, glucose, and insulin, as well as IGF-I and IGFBP-3 were measured. IGF-I concentrations were also expressed by IGF-I SDS, according to the reference data [26].

For the calculation of IGF-I/IGFBP-3 molar ratio, the following molecular masses were used: 7.5 kDa for IGF-I and 42.0 kDa for IGFBP-3. For IGF-I/IGFBP-3 molar ratio, the cutoff point was established at the median values.

According to the results, the group of GHD children was divided into two subgroups: GHD children with low IGF-I bioavailability (*n* = 13; IGF-I/IGFBP-3 molar ratio below the cutoff level) and with normal IGF-I bioavailability (*n* = 13; IGF-I/IGFBP-3 molar ratio equal or higher than the cutoff point). Based on the results of fasting glucose and insulin concentration, the insulin resistance (IR) index (IRI) HOMA was calculated [27].

The control group consisted of 39 healthy children (12 girls and 27 boys), aged 5.1 to 16.6 years (mean ± SD: 10.87 ± 3.12 years), with normal body height and normal body weight. In that group of children, fasting total ghrelin, leptin, adiponectin, resistin, lipids, glucose, and insulin, as well as IGF-I and IGFBP-3 concentrations were assessed.

Growth hormone levels were measured using the immunometric method. The measurements were performed by Immulite, DPC assay kits, calibrated to the WHO IRP 98/574 standard set of the following sensitivity level: 0.01 ng/ml, range: up to 40 ng/ml, conversion index: ng/ml × 2.6 = mIU/l, the intra-assay CV: 5.3–6.5%, and interassay CV: 5.5–6.2%.

Both IGF-I and IGFBP-3 concentrations were assessed by the Immulite, DPC assays; WHO NIBSC 1st IRR 87/518 standard was applied, with the analytical sensitivity of 20 ng/ml, calibration range up to 1600 ng/ml, the intra-assay CV: 3.1–4.3%, and interassay CV: 5.8–8.4%. The assay for IGFBP-3 assessment was calibrated to WHO NIBSC Reagent 93/560 standard, with analytical sensitivity 0.02 *μ*g/ml, the calibration range up to 426 *μ*g/ml, the intra-assay CV: 3.5–5.6%, and the total CV: 7.5–9.9%.

The total ghrelin concentration was measured using the radioimmunometric method with Millipore assay kits of the following sensitivity level: 100–10.000 pg/ml, the intra-assay CV: 3.3–10.0%, and interassay CV: 14.7–17.8%.

The leptin, resistin, and adiponectin concentrations were measured using the Millipore ELISA kit (Linco Research). The sensitivity level, intra-assay CV, and interassay CV were as follows: 0.5–100 ng/ml, 1.4–4.9%, and 1.3–8.6% for leptin; from 0.16 ng/ml, 3.2–7.0%, and 7.1–7.7% for resistin; and from 0.78 ng/ml, 7.4%, and 2.4–8.4% for adiponectin, respectively.

Plasma insulin concentration was measured using the DRG ELISA kit; sensitivity level 1.76–100 *μ*IU/ml, the intra-assay CV: 1.8–2.6, and interassay CV: 2.9–6.0. Plasma glucose concentration was determined with the enzymatic method, with the use of hexokinase.

Shapiro-Wilk's test was used to assess the distribution of the variables. A chi-square test and a one-way ANOVA were applied for statistical analysis, with the subsequent use of a post hoc test, in order to statistically assess differences between individual pairs of groups. Correlations were evaluated using Pearson's test. Statistically significant differences were accepted when *p* value was below 0.05.

## 3. Results

First, the data of the GHD group and of the control group were analysed (Table 1). It was not surprising that the children with GHD were significantly shorter (see HSDS values) and heavier (see BMI SDS for HA values) than children from the controls.

In the group of children with GHD, significantly higher levels of ghrelin from the controls (1352.03 ± 715.75 versus 1064.10 ± 493.39 pg/ml) and leptin (13.09 ± 11.72 versus 7.74 ± 9.14 ng/ml) but lower levels of resistin (9.44 ± 2.90 versus 12.68 ± 6.33 ng/ml) were recorded. The adiponectin concentrations were similar in both groups. Significantly higher concentrations of triglycerides (84.71 ± 34.17 versus 63.47 ± 26.64 mg/dl) in GHD than in the controls were observed, whereas there was no difference between the groups as regards the concentrations of cholesterol and its fractions. Fasting glucose and insulin concentrations, as well as IRI HOMA did not differ between the groups.

We found that IGF-I SDS value was not correlated with any of the analysed parameters in the studied group of children (GHD and controls together, data not shown), while IGF-I/IGFBP-3 molar ratio was negatively correlated with ghrelin (*r* = −0.35, *p* < 0.05) and positively with triglycerides (*r* = +0.4, *p* < 0.05) and insulin (*r* = +0.41, *p* < 0.05) concentrations, as well as with IRI HOMA (*r* = +0.36, *p* < 0.05) (Table 2). In turn, among children with GHD, we confirmed a negative correlation between the IGF-I/IGFBP-3 molar ratio and both ghrelin (*r* = −0.34, *p* < 0.05) and adiponectin (*r* = −0.41, *p* < 0.05) and a positive one between IGF-I/IGFBP-3 molar ratio and both insulin (*r* = +0.48, *p* < 0.05) and IRI HOMA (*r* = +0.41, *p* < 0.05). Also, we observed a negative correlation between IGF-I/IGFBP-3 molar ratio and HDL-cholesterol concentration in GHD children (*r* = −0.59, *p* < 0.05) (Table 2).

Thus, the GHD children—according to values of IGF-I/IGFBP-3 molar ratio—were divided into two subgroups: lower IGF-I bioavailability and higher IGF-I bioavailability (see Materials and Methods).

It was found that the children from GHD-lower-IGF-I bioavailability group were significantly slimmer (even after correction of BMI SDS values for HA) than the children from GHD-higher-IGF-I bioavailability group. The concentrations of cholesterol and its fractions did not differ between groups. Furthermore, we observed a significantly lower triglyceride (68.14 ± 28.34 versus 96.30 ± 34.32 mg/dl) and insulin (5.92 ± 4.43 versus 9.07 ± 3.63 uIU/ml) concentrations, as well as IRI HOMA (1.36 ± 1.04 versus 1.93 ± 0.94) in the GHD group with lower IGF-I bioavailability than in the GHD group with higher IGF-I bioavailability. Also, significantly higher ghrelin (1648.95 ± 657.60 versus 1005.64 ± 640.44 pg/ml) and adiponectin (23.72 ± 6.3 versus 15.06 ± 6.18 ng/ml) concentrations in the group with lower IGF-I bioavailability than in the group with higher IGF-I bioavailability were observed. In turn, both leptin and resistin levels were lower in children with lower IGF-I bioavailability than in those with higher IGF-I bioavailability; however, the differences did not reach a border of statistical significance (Table 3).

The maximal GH secretion during stimulation test after clonidine administration and during nocturnal profile was similar in both groups. Interestingly, we observed a significantly lower maximal concentration of GH during stimulation test after glucagon administration in the lower IGF-I bioavailability than in the higher IGF-I bioavailability group (4.50 ± 1.56 versus 7.33 ± 2.85 ng/ml) (Table 3).

## 4. Discussion

The results of our study showed that the GHD children group was not homogeneous as regards the metabolic profile, nutritional state, and IGF-I bioavailability. On the one hand, it included children with GHD of both the pituitary and hypothalamic origins with different etiology; on the other, it is well known that IGF-I concentration depends not only on GH secretion but also on the nutritional status of children (which in turn is conditioned by many different factors). Moreover, concentrations of certain neuropeptides (i.e., ghrelin, leptin, and insulin) also affect IGF-I level [9, 10, 21].

In our study, the worse metabolic profile characterised those with bigger body mass and higher IGF-I bioavailability and lower ghrelin and adiponectin concentrations. It is not clear what is the cause and what is the effect of disturbances.

It is well known that adult patients with GHD show a tendency towards metabolic disorders (higher body mass, unfavourable lipids, and increased leptin and insulin concentrations) and that they return to normal levels following GH treatment [28, 29]. However, the metabolic profile of untreated children with GHD varies in individual reports [4–8]. In Gleeson et al.'s study [4], the lipid profile was disturbed at baseline in GHD children, while abnormal body composition was only observed in older subjects in late puberty. Also, Capalbo et al. reported [5] higher waist to hip ratio, triglycerides, total cholesterol, LDL-cholesterol, and leptin in GHD children compared to controls, whereas no differences in adiponectin concentration were found. Next, Ciresi et al. [6] observed that total and LDL cholesterol were higher in GHD children than in controls, whereas HDL cholesterol, triglycerides, insulin, HOMA-IR, leptin, and adiponectin were similar. López-Siguero et al. [7] confirmed that adiponectin concentration was markedly elevated in GHD children when compared to controls. Similar observations were presented by Meazza et al. [8]. In the group of GHD children analysed by these authors, higher concentrations of both adiponectin and resistin were observed. It should be stressed that there are no unequivocal results in the studies of the aforesaid authors indicating that children with GHD are thicker than those of the control group [5, 6], and therefore, excessive body weight is not a permanent feature of GHD in children.

Our results for the whole group of children with GHD are not fully consistent with the results obtained by the authors mentioned above. We decided to test the hypothesis that differences in the metabolic profile in GHD children observed by different groups of researchers might depend on the IGF-I bioavailability and, in consequence, altered compensatory synthesis of these hormones which play an important role in glucose and lipid metabolism (i.e., ghrelin, adiponectin, resistin, and leptin).

Analysing our results, we found worse metabolic profile (higher BMI, higher triglycerides and insulin concentrations, and higher IRI HOMA) in GHD children with higher IGF-I bioavailability than in GHD children with lower IGF-I bioavailability. The explanation of this phenomenon is not fully clear. It seemed to us that those individuals with GHD who have the lowest bioavailability of IGF-I should present the most severe GHD symptoms. In adults with GHD, strong negative correlations between GH peak concentration and visceral adiposity index (VAI), as well as between IGF-I level and VAI have been proved [29]. VAI takes into account such parameters as BMI and waist circumference, as well as both triglyceride and HDL-cholesterol concentrations. It is a reliable marker for adipose tissue function and distribution. However, generally in children, the correlation between GH peak and IGF-I concentrations is not as obvious as in adults [10, 30].

It is generally accepted that IGF-I is a crucial value for promoting the anabolic effects of GH. However, it should be noted that the diagnostic schedule of GHD deficiency is based on the results of GH-stimulating tests, and in some children with GHD, the IGF-I concentrations were within the reference range. Although IGF-I concentration has widely been used for monitoring GH dosing, it is still not an optimal marker for the diagnosis of GHD [31]. The mechanisms by which GH regulates substrate metabolism are not fully understood; perhaps, some of them occur indirectly through IGF-I or antagonism of insulin action [2].

We have taken into consideration the possibility that in the group of GHD children with better IGF-I bioavailability, the unfavourable metabolic profile (higher triglycerides and insulin concentrations) may be related to higher body mass, which has been observed in that group of children. However, some aspects of this issue should also be discussed. First, although children in the group with lower IGF-I bioavailability were significantly slimmer, we did not find any correlation between IGF-I concentration (also IGF-I SDS and IGF-I/IGFBP-3 molar ratio) and BMI SDS in our group of GHD children. Second, it is well known that in children with obesity, lower GH secretion during stimulating tests in comparison to health population are observed [32, 33]. This effect is probably the result of somatostatin hypersecretion and decreased ghrelin production [34], as well as hyperinsulinism and elevated concentrations of free fatty acids [32]. Although separate reference data for GH response to most provocative stimuli in obesity are not available, some authors propose to define BMI-specific cutoff points for GHD-diagnosing tests [35, 36]. However, in the group of children analysed by us, we did not have any child with BMI higher than 2.0 SDS. Furthermore, one of the qualification criteria into the GHD group was too slow height velocity, which was not observed in children with simple obesity related to hyperalimentation. Thus, the suspicion that children from the group of GHD-higher-IGF-I bioavailability were chosen incorrectly, due to false-negative GH-stimulating tests results, does not seem justified.

Among the GHD group, the children with better, as well as with worse nutritional state were identified. Thus, it is possible that in the GHD children with better IGF-I bioavailability and greater body mass, the metabolic profile typical for adult GHD is observed. In turn, in the GHD children with low IGF-I bioavailability and lower body mass, their thinness (for various reasons: silent gastrointestinal diseases, decreased level of neuropeptide-stimulating orexigenic axis, and children with failure to thrive) is accompanied by GHD (and is superimposed on GHD), which results in even more reduced IGF-I production. However, in these cases, the higher adiponectin concentrations were recorded. Perhaps, it was the reason that in that group, a much better metabolic profile was presented (atypical for classic GHD).

It should also be emphasized that children with chronic diseases and those with undiagnosed gastrointestinal tract complaints were excluded from the study group. This eliminates other GHD causes of secondary IGF-I deficiency, especially those observed in children with malnutrition.

The analysis of our data showed that in GHD children with low IGF-I bioavailability, the ghrelin and adiponectin concentrations were significantly higher than in GHD children with higher (better) IGF-I bioavailability. We had reported a higher ghrelin concentration in GHD than in controls in our earlier work [11] and the negative correlation between ghrelin and IGF-I/IGFBP-3 molar ratio [10]. Indeed, a higher ghrelin concentration was observed only in those children with GHD in whom low IGF-I bioavailability was confirmed. In the group of GHD children with better IGF-I bioavailability, the ghrelin concentrations were similar to controls. It is known that both GH and ghrelin are hormones exerting a significant effect on the adipose tissue metabolism but they act differently: GH stimulates lipolysis and reduces the fat accumulation, while ghrelin decreases lipolysis and metabolism which implies that ghrelin can induce an increase in adipose tissue and weight gain [37]. According to the studies of other authors, GH causes relative insulin resistance by reduced peripheral glucose uptake, while ghrelin reduces glucose-stimulated insulin secretion, leading to the deterioration of glucose tolerance [1, 16, 38, 39].

Therefore, in children with GH deficiency and an excess of ghrelin secretion, a tendency towards metabolic disorders should be suspected. In contrast, in the group of children with GHD and higher ghrelin concentration that we analysed, the metabolic profile was better than in children with GHD and lower ghrelin concentration.

Further, it seems that ghrelin is not the cause of obesity or leanness, but rather one aspect of a compensatory mechanism that maintains body energy homeostasis [40]. The diet-induced obesity suppressed the neuroendocrine ghrelin system by decreasing ghrelin production in the stomach, as well as by ghrelin resistance in arcuate neuropeptide Y/Agouti-related peptide (NPY/AgRP) neurons [41]. It was proved that in obese patients, ghrelin concentration is reduced, while in patients with anorexia nervosa or malnourished subjects, it was increased [42]. On the other hand, the GH receptor deficiency blunts the stimulatory effect of ghrelin on feeding and lipid production in mice [43]. It appears that in GHD children with lower IGF-I secretion and higher ghrelin production, the ghrelin effect on lipids may also be disturbed. As regards insulin, it is well known that the high circulating ghrelin level is associated with lower insulin resistance in the general population and exogenously infused ghrelin reduces insulin secretion in healthy humans [44]. The lower insulin concentration and lower IRI HOMA in GHD children with higher ghrelin were also observed by us in the present study.

In turn, as regards adiponectin, its concentration was elevated in the GHD group when we compared it to the controls. However, among children from the GHD group, the higher adiponectin concentration was observed in those with lower IGF-I bioavailability than in those with higher IGF-I bioavailability. It is well known that adiponectin has a beneficial effect on the metabolic profile in children and adults [20, 22]. Adiponectin directly induces GH secretion from somatotropes [45]. Previously, Oliviera et al. [22] have documented high adiponectin levels in GHRH resistance patients with GHD and very low IGF-I. The authors suggested that high adiponectin production could be a compensatory mechanism to the lack of GHRH action. Also in GH receptor-deficient mice, an elevated adiponectin levels were observed [46]. Regardless of the mechanism by which adiponectin is increased in our GHD-low-IGF-I group, it is possible that the better metabolic profile is mainly related to higher adiponectin levels.

As regards leptin and resistin, we found a significantly higher level of leptin and lower of resistin in children with GHD in comparison to controls. However, after dividing the GHD group into subgroups, distinguished on the basis of IGF-I bioavailability, we did not find significant differences between them. It has been showed that both leptin and resistin concentrations are not important for differences in the metabolic profile observed in both subgroups of GHD children.

Summing up, GHD children are a heterogeneous group as regards pathogenesis of the diseases. Differences in bioavailability of IGF-I cause the alter secretion of ghrelin and adiponectin which probably determines the differences in the metabolic profile of GHD patients. However, further studies are necessary to explain the relationships among ghrelin, adiponectin, GH/IGF-I secretion, and orexigenic axis action. The effect is probably related to the overlapping of several elements.

The purpose of our considerations was not to overturn the thesis that GH treatment in GHD (which obviously results in an increase of IGF-I concentration) does improve metabolic processes. The metabolic benefits of GH therapy, both in adults and in children, are undeniable. The aim of our study was to present the different possible variants of GHD in children before treatment, resulting from different etiology of disease, or a coexistence of disorders overlapping GHD.

## 5. Conclusion

In GHD children, the metabolic profile is related to IGF-I bioavailability and nutritional status, as well as ghrelin and adiponectin production. In GHD children in whom the lower bioavailability of IGF-I is observed, the better metabolic profile is noted. The lower the IGF-I bioavailability, the better metabolic profile is observed. It may depend on higher adiponectin related to good insulin sensitivity; however, unfavourable effects of ghrelin action on lipids were not observed by us.

## Figures and Tables

**Table 1:** Auxological data and hormonal test results in GHD children and in controls.

Group	GHD	Controls
Number of children (girls/boys)	26 (11/15)	39 (12/27)
Chronological age (years)	10.91 ± 2.55	10.87 ± 3.12
Height SDS	−2.53 ± 0.56^a^	−0.58 ± 1.11^a^
BMI (kg/m^2^)	18.51 ± 3.64	18.23 ± 3.51
BMI SDS for CA	0.37 ± 1.47	0.15 ± 1.12
BMI SDS for HA	0.94 ± 1.70^b^	0.31 ± 1.14^b^
IGF-I (ng/ml)	184.18 ± 85.61	208.13 ± 123.77
IGF-I SDS	−1.21 ± 0.90^b^	−0.29 ± 1.03^b^
IGFBP-3 (*μ*g/ml)	4.49 ± 1.28	4.65 ± 1.10
IGF-I/IGFBP-3 molar ratio	0.25 ± 0.12	0.24 ± 0.14
Ghrelin (pg/ml)	1352.03 ± 715.75^b^	1064.10 ± 493.39^b^
Leptin (ng/ml)	13.09 ± 11.72^b^	7.74 ± 9.14^b^
Adiponectin (ng/ml)	19.18 ± 12.58	17.71 ± 8.12
Resistin (ng/ml)	9.44 ± 2.90^b^	12.68 ± 6.33^b^
Triglycerides (mg/dl)	84.71 ± 34.17^b^	63.47 ± 26.64^b^
Total cholesterol (mg/dl)	171.70 ± 33.91	162.00 ± 31.60
LDL cholesterol (mg/dl)	96.63 ± 31.43	86.79 ± 23.96
HDL cholesterol (mg/dl)	60.19 ± 14.21	62.84 ± 15.65
Glucose 0′ (mg/dl)	84.29 ± 8.01	85.10 ± 6.24
Insulin 0′ (uIU/ml)	7.67 ± 4.20	6.93 ± 4.16
IRI HOMA	1.70 ± 0.99	1.47 ± 0.93

Data are presented as the means ± SD. GHD: growth hormone deficiency; SDS: standard deviation score; BMI: body mass index; CA: chronological age; HA: height age; IGF-I: insulin-like growth factor I; IGFBP-3: insulin-like growth factor binding protein 3; LDL-cholesterol: low density lipoprotein-cholesterol; HDL-cholesterol: high density lipoprotein-cholesterol; IRI HOMA: insulin resistance index according to homeostasis model assessment. In the individual rows of the table, the variables designated with the same letters differ significantly from each other with ^a^*p* < 0.00000 and ^b^*p* < 0.05. One-way analysis of variance (ANOVA) was used for group comparison.

**Table 2:** Correlation coefficients between IGF-I/IGFBP-3 molar ratio and individual analysed parameters in the total group of children and separately in the GHD group.

Correlation between IGF-I/IGFBP-3 molar ratio and:	Total group (GHD and controls)	GHD group
Height SDS	+0.01	+0.16
BMI SDS for HA	+0.26	+0.24
Ghrelin	−0.35^∗^	−0.34^∗^
Leptin	+0.29	+0.34
Adiponectin	−0.15	−0.41^∗^
Resistin	−0.24	0.0
Triglycerides	+0.4^∗^	+0.59^∗^
Cholesterol	+0.22	+0.05
LDL-cholesterol	+0.16	+0.08
HDL-cholesterol	−0.04	−0.59^∗^
Glucose	+0.18	+0.2
Insulin	+0.41^∗^	+0.48^∗^
IRI HOMA	+0.36^∗^	+0.41^∗^

In the individual rows of the table, the correlation coefficients designated with the asterisks differ significantly from each other.

**Table 3:** Auxological data and hormonal tests results in GHD children with lower IGF-I bioavailability and in GHD children with higher IGF-I bioavailability.

	GHD children with lower IGF-I bioavailability	GHD children with higher IGF-I bioavailability
Number of children (girls/boys)	14 (6/8)	12 (5/7)
Chronological age (years)	10.24 ± 2.37	11.99 ± 2.42
Height SDS	−2.42 ± 0.48	−2.66 ± 0.63
BMI (kg/m^2^)	16.54 ± 2.53^b^	20.65 ± 3.51^b^
BMI SDS for CA	−0.21 ± 1.43^d^	0.99 ± 1.31^d^
BMI SDS for HA	0.13 ± 1.44^c^	1.81 ± 1.55^c^
GH_max_ after clonidine (ng/ml)	7.06 ± 1.88	7.64 ± 1.80
GH_max_ after glucagon (ng/ml)	4.50 ± 1.56^d^	7.33 ± 2.85^d^
GH_max_ during nocturnal profile	8.83 ± 5.41	7.34 ± 3.53
IGF-I (ng/ml)	133.33 ± 54.22^a^	243.50 ± 77.79^a^
IGF-I SDS	−1.79 ± 0.63^b^	−0.67 ± 0.79^b^
IGFBP-3 (*μ*g/ml)	4.46 ± 1.17	4.52 ± 1.42
IGF-I/IGFBP-3 molar ratio	0.16 ± 0.07^b^	0.32 ± 0.11^b^
Triglicerides (mg/dl)	68.14 ± 28.34^d^	96.30 ± 34.32^d^
Total cholesterol (mg/dl)	169.89 ± 31.15	173.18 ± 37.46
LDL cholesterol (mg/dl)	105.43 ± 22.16	89.78 ± 36.92
HDL cholesterol (mg/dl)	62.17 ± 13.95	59.00 ± 14.98
Ghrelin (pg/ml)	1648.95 ± 657.60^d^	1005.64 ± 640.44^d^
Leptin (ng/ml)	9.82 ± 8.20	16.06 ± 13.93
Adiponectin (ng/ml)	23.72 ± 6.3^d^	15.06 ± 6.18^d^
Resistin (ng/ml)	8.70 ± 2.51	10.11 ± 3.18
Glucose 0′ (mg/dl)	84.86 ± 4.49	83.90 ± 10.00
Insulin 0′ (uIU/ml)	5.92 ± 4.43^d^	9.07 ± 3.63^d^
IRI HOMA	1.36 ± 1.04^d^	1.93 ± 0.94^d^

Data are presented as the means ± SD. GHD: growth hormone deficiency; SDS: standard deviation score; BMI: body mass index; CA: chronological age; HA: height age; IGF-I: insulin-like growth factor I; IGFBP-3: insulin-like growth factor binding protein 3; LDL-cholesterol: low density lipoprotein-cholesterol; HDL-cholesterol: high density lipoprotein-cholesterol; IRI HOMA: insulin resistance index according to homeostasis model assessment. In the individual rows of the table, the variables designated with the same letters differ significantly from each other with ^a^*p* < 0.0005, ^b^*p* < 0.005, ^c^*p* < 0.01, and ^d^*p* < 0.05. One-way analysis of variance (ANOVA) was used for group comparison.
